# Cardiac tamponade masquerading as gastritis: a case report

**DOI:** 10.1186/1752-1947-8-264

**Published:** 2014-07-30

**Authors:** Abuzaid Ahmed, Tella Sri Harsha, Tantoush Hamza, Ameri Allen, Elkhashab Mohamed

**Affiliations:** 1 Creighton University Medical Center, 601 30th street, 5th floor, Omaha, NE 68131, USA

**Keywords:** Echocardiography, Pericardial effusion, Pulsus paradoxus, Tamponade

## Abstract

**Introduction:**

Pericardial effusion and cardiac tamponade can develop in patients with virtually any condition that affects the pericardium. A high index of suspicion with proper diagnostic scheme can lessen the concomitant morbidity and mortality. Although cardiac tamponade mimics many medical conditions, internists and primary care physicians should be aware of the physiological and clinical aspects of the disease spectrum.

**Case presentation:**

A 31-year-old Caucasian man, with no significant past medical history, presented to our emergency room with acute upper abdominal heaviness of 2 hours’ duration after drinking excessive amounts of alcohol in a short period of time (binge drinking). The coexistence of recent alcohol binge drinking and nonspecific abdominal complaints usually presume a diagnosis of gastritis in our daily encounters in the absence of hepatic, biliary or pancreatic derangements. We present a case in which the presenting abdominal pain turned out to be related to cardiac tamponade.

**Conclusions:**

Cardiac tamponade is a sort of cardiogenic shock and is a medical emergency. Clinicians should understand the cardiac tamponade physiology, especially in cases without large pericardial effusion, and correlate the signs of clinical tamponade together with the echocardiographic findings. Drainage of cardiac tamponade is life-saving. A high index of suspicion with proper diagnostic arcades lessens the concomitant morbidity and mortality.

## Introduction

Pericardial effusion and cardiac tamponade can develop in patients with virtually any condition that affects the pericardium, including post-pericarditis, malignancies, chronic renal failure, thyroid disease, autoimmune disease, and traumatic and idiopathic causes. Symptoms and signs lack both sensitivity and specificity. A high index of suspicion with proper diagnostic scheme can lessen the concomitant morbidity and mortality. Internists and primary care physicians should be aware of the physiological and clinical aspects of the disease spectrum.

## Case presentation

A 31-year-old Caucasian man, with no significant past medical history, presented to our emergency room (ER) with an acute upper abdominal heaviness of 2 hours’ duration. He was drinking that night at a bar with friends and drank approximately 500ml of vodka. He vomited later while at home and had chest heaviness before arrival. In the ER, he was confused and not able to give exact details about his pain. With this history and presentation, gastritis was suspected. After treatment with an antiemetic and analgesics, he was able answer our questions. He quantified his pain as 5/10 in the upper abdominal and precordial regions, dull in nature and improved on forward posture. On further questioning, he related flu-like symptoms a week ago and for the last 2 days he had a sharp chest pain limiting his exertion. A trial of ibuprofen did alleviate some of his symptoms and he did not seek any medical advice initially.

**Figure 1:**
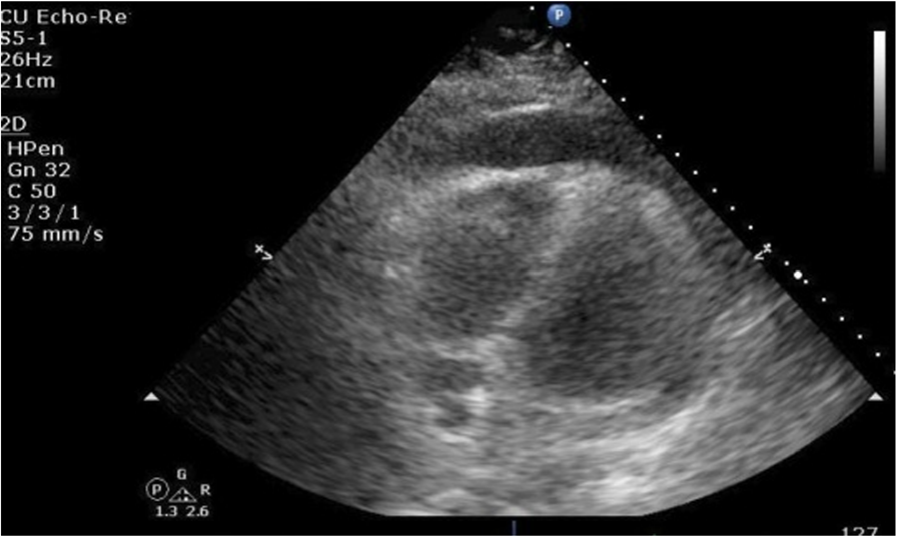
**A transthoracic echocardiography showing the heart surrounded by moderate pericardial effusion seen as echo-free space more than 12mm with evidence of right ventricular collapse.** Apical four-chamber view.

**Figure 2:**
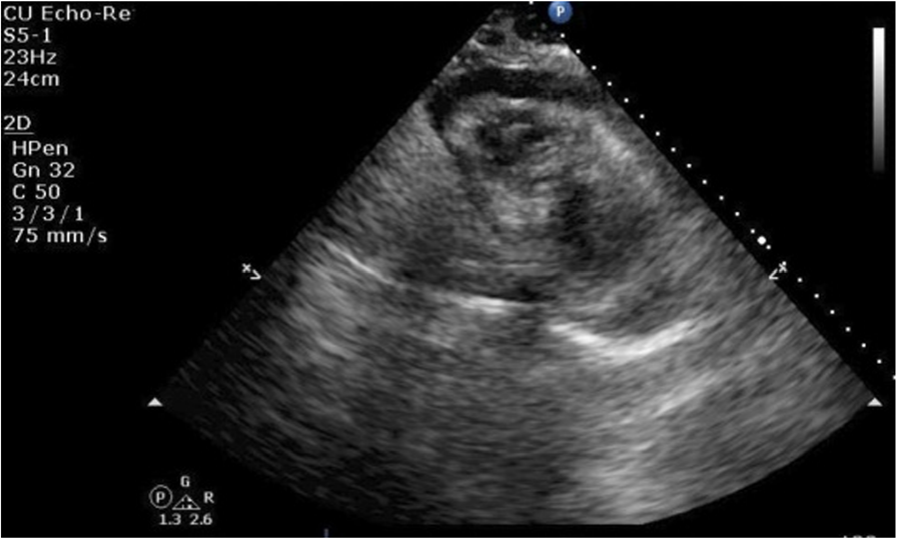
**A transthoracic echocardiography showing the heart surrounded by moderate pericardial effusion seen as echo-free space more than 12mm with evidence of right ventricular collapse.** Right ventricular short-axis view.

**Figure 3:**
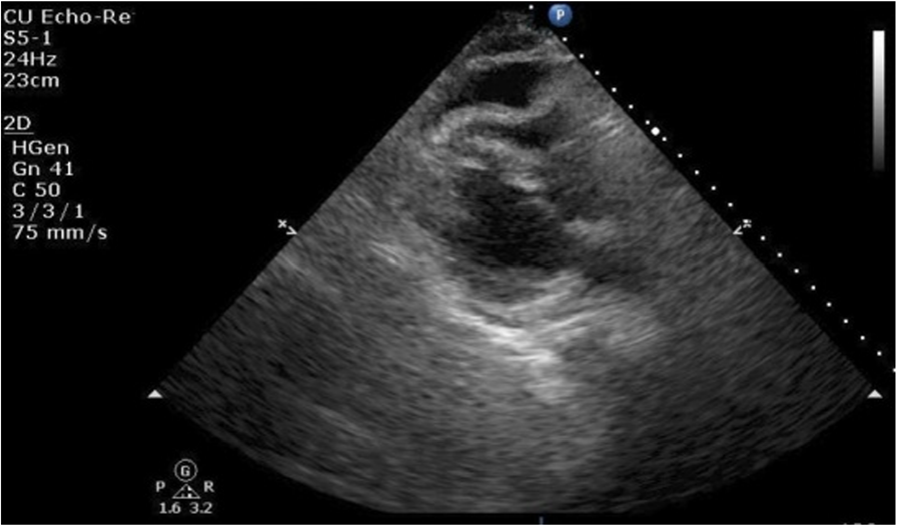
**A transthoracic echocardiography showing the heart surrounded by moderate pericardial effusion seen as echo-free space more than 12mm with evidence of right ventricular collapse.** Parasternal long-axis view.

**Figure 4:**
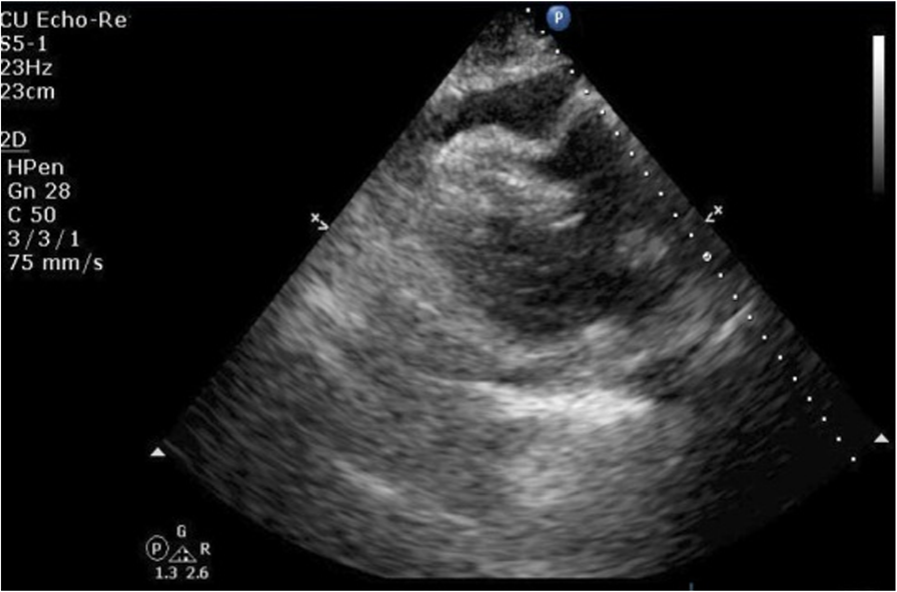
**A transthoracic echocardiography showing the heart surrounded by moderate pericardial effusion seen as echo-free space more than 12mm with evidence of right ventricular collapse.** Parasternal long-axis view with right ventricular collapse with fluid width about 1.44cm.

**Figure 5:**
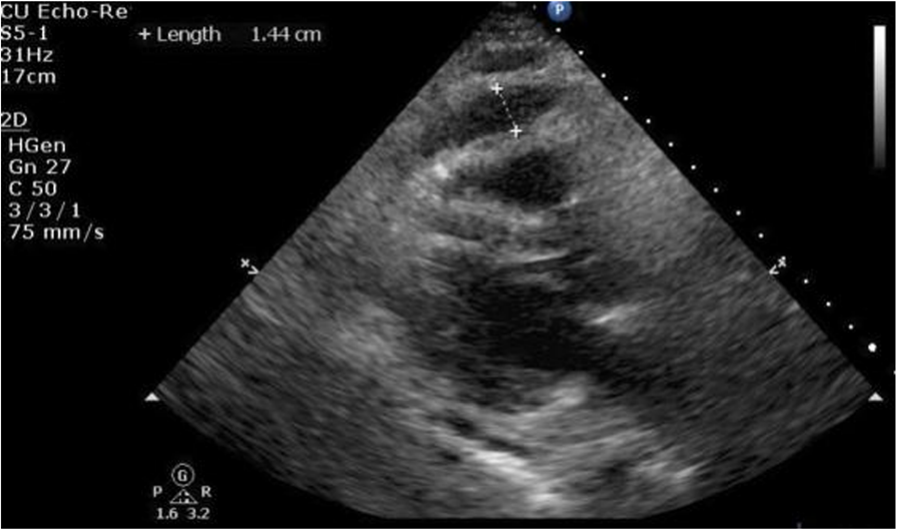
**A transthoracic echocardiography showing the heart surrounded by moderate pericardial effusion seen as echo-free space more than 12mm with evidence of right ventricular collapse.** Parasternal long-axis view with right ventricular collapse with fluid width about 1.44cm.

Initial vitals revealed a blood pressure of 134/96mmHg, pulse of 79/minute, respiratory rate of 20/minute and temperature of 37.5°C (99.5°F). On physical examination there were muffled heart sounds and slightly raised veins. Pulsus paradoxus of 18mmHg was noted. His abdomen was soft and non-tender on palpation.His history and physical examination were suspicious for pericardial effusion. Laboratory investigation was significant for a white blood cell count of 20.0K/uL with mild lymphocytosis. Liver function tests and pancreatic enzymes were within normal limits. A chest X-ray in posteroanterior projection and abdominal films were unremarkable. An electrocardiogram (EKG) showed low voltage waves in all leads and P-R depression in lead II. Transthoracic echocardiography (TTE) showed a moderate pericardial effusion compromising the right ventricular filling (Figures 1, 2, 3, 4 and 5).

He eventually had a pericardial window as it was problematic to reach the pericardial sac with a transthoracic needle. After the procedure he felt better and he was started on ibuprofen 800mg three times a day for 10 days. Two weeks later, he was seen in the out-patient clinic and TTE revealed a normal pericardial sac with no effusion.

## Discussion

In our patient, the vague presentation of abdominal pain was mimicking gastritis. A prompt physical examination and high index of suspicion had solved our dilemma, and potential sequels were prevented. We are writing this case to re-emphasize the importance of the physical examination in the field of medicine and to alert physicians about the unusual presentation of a common disease which can be overlooked.

A presumptive diagnosis of acute viral pericarditis leading to pericardial tamponade was established. Cardiac tamponade is a medical emergency. Timely diagnosis and intervention are important to prevent mortality.

Acute pericarditis is a clinical syndrome caused by inflammation of the pericardium and is associated with chest pain, a friction rub and characteristic electrocardiographic changes [1]. Pericardial effusion is a fluid collection in the pericardial space; depending on the underlying etiology and rate of accumulation, the clinical presentations may range from being asymptomatic to a life-threatening scenario [1-4].

Large pericardial effusion may be found unexpectedly without significant elevation of intrapericardial pressure and is usually asymptomatic, whereas rapidly accumulating effusions may result in compressive physiology and hence tamponade, characterized by a progressive limitation of ventricular diastolic filling and a reduction in cardiac output [2,3,5].

Acute cardiac tamponade occurs within minutes, due to trauma and rupture of the heart or great vessels, resembling cardiogenic shock that requires urgent drainage [2,6]. Subacute events, as in our patient, are usually less dramatic and occur over days to weeks and usually due to non-traumatic causes [1-3].

Although cardiac tamponade is considered a clinical diagnosis; findings like hypotension, tachycardia, elevated jugular venous pressure, and pulsus paradoxus, are known to have limited sensitivity and specificity [2,7]. Every effort should be made to narrow the wide range of possible etiologies, especially in the acute life-threating tamponade, a variant of cardiogenic shock [8,9].

Large effusion may be associated with muffled heart sounds [1]. Dullness to percussion and bronchial breathing below the left scapular angle is rarely appreciated (Ewart’s sign). Sinus tachycardia and hypotension are signs of hemodynamic compromise. Tachycardia is usually absent in hypothyroid or uremic individuals. In advanced cases where pulseless electrical activity can occur and heroin, a scenario of cardiac arrest can be puzzling, where urgent tapping can be life-saving with effortless external chest compression efforts [10,11].

Careful examination of pulse may anticipate the presence of pulsus paradoxus, with more than 10mmHg drop in systolic pressure due to impairment of left ventricular filling by the displaced septum during right ventricular filling and consequent drop in systolic pressure, a phenomenon known as ventricular interdependence [1]. Evaluation for pulsus paradoxus should always be performed during normal respiration because deep inspiration may render a false positive finding [11]. Physicians should be aware of other conditions in which pulsus paradoxus may be present without cardiac tamponade as obstructive airway diseases, such as marked obesity, massive pulmonary embolism, profound hypovolemic shock, severe pectus excavatum, bilateral pleural effusion, right atrial mass, right ventricular myocardial infarction and tension pneumothorax [2,3]. Pulsus paradoxus is not specific for cardiac tamponade [1-3,12]. Pulsus paradoxus may be absent in the presence of dehydration and in conditions with raised ventricular diastolic pressures as chronic hypertension or coexisting atrial septa defect or significant aortic regurgitation.

Beck’s triad describes the combination of venous distension, distant heart sounds and absolute or relative hypotension. Pericardial rub may be audible, particularly in inflammatory pericarditis [11] and cardiac apical impulse could be reduced or absent [1].

The classic EKG finding in large effusions consists of low voltage tracing [13]. Electrical alternans is a fairly specific sign of massive effusion [13-15]. For cardiomegaly to be evident in chest radiography, a minimum of 200 to 250mL of fluid has to be accumulated in the pericardial sac [14]. If possible, lateral chest films should be attempted to detect large effusions [14].

Cardiac tamponade is not an “all-or-none” occurrence, but rather a continuum of findings [16]. Clinicians should correlate the echocardiographic signs of tamponade (right ventricle collapse, right atrium collapse, respiratory variation of the mitral and tricuspid flow, and inferior vena cava plethora) with the symptoms and signs in each tamponade case [5,8,17].

## Conclusions

Pericardial effusion can develop in patients with virtually any condition that affects the pericardium, including acute-, sub-acute pericarditis, malignancies, pulmonary tuberculosis, chronic renal failure, thyroid disease, autoimmune disease, or iatrogenic and idiopathic causes. Symptoms and signs lack both sensitivity and specificity. Transthoracic echocardiography is the most important tool for diagnosis, grading, drainage and follow-up. Cardiac tamponade is a sort of cardiogenic shock and a medical emergency. Clinicians should understand the cardiac tamponade physiology, especially in cases without large pericardial effusion and correlate the signs of clinical tamponade together with the echocardiographic findings. Drainage of cardiac tamponade is life-saving. A high index of suspicious with proper diagnostic arcades lessens the concomitant morbidity and mortality.

## Patient’s perspective

I write the following to provide assistance to the case report written about my hospitalization. I have no medical knowledge or background so I only write from my own perspective and experience.

Initially, I was told that I had gastritis after alcohol drinking. When the internal medicine team was called in for admission I was more awake and was able to give history. With that history, detailed physical exam, and EKG findings I was told that I am suspected to have what is called cardiac tamponade that was confirmed on TTE. A detailed explanation about my condition was given. On the same day, I felt better after a pericardial window which drained my suffering sac. I was really grateful for the timely diagnosis and intervention. I could say that alcohol had saved my life otherwise I would not have come to the hospital!, but I am not drinking again.

## Consent

Written informed consent was obtained from the patient for publication of this case report and accompanying images. A copy of the written consent is available for review by the Editor-in-Chief of this journal if needed.

## Abbreviations

EKG: Electrocardiogram; ER: Emergency room; TEE: Transthoracic echocardiography.

## Competing interests

The authors declare that they have no competing interests.

## Authors’ contributions

AAh analyzed and interpreted the patient data regarding the presentation and hospital course. TH, AAl, EM were major contributors in writing the manuscript. All authors read and approved the final manuscript.
